# Depression and breast cancer: research progress and prospects from an interdisciplinary perspective

**DOI:** 10.3389/fimmu.2026.1760846

**Published:** 2026-06-02

**Authors:** Kang He, Huiyang Tang, Cunjun Mai, Yongzhen Li, Dongmin Yu

**Affiliations:** 1 Department of Breast Disease Comprehensive Center, First Affiliated Hospital of Gannan Medical University, Ganzhou, China; 2 First Clinical Medical College, Gannan Medical University, Ganzhou, China

**Keywords:** breast cancer, comorbidity, depression, immunosuppression, neuroinflammation, women’s health

## Abstract

Depression and breast cancer are two major health concerns in the medical field today. Not only do they mutually influence the risk of onset and disease progression, but they also collectively lead to poorer clinical outcomes and reduced quality of life. This review elaborates on how depression, through the overactivation of the hypothalamic-pituitary-adrenal (HPA) axis, may be associated with persistently elevated levels of glucocorticoids (GCs). This, in turn, causes dysregulation of the sex hormone axis, prolactin imbalance, and insulin resistance, collectively promoting the initiation and progression of breast cancer. Meanwhile, the depressive state may suppresses the function of immune cells such as T cells and natural killer (NK) cells, and promotes the release of pro-inflammatory cytokines, thereby impairing immune surveillance and creating a favorable environment for tumor growth. Furthermore, imbalances in tryptophan metabolism and gut microbiota dysbiosis exacerbate neuroimmune dysregulation, forming a vicious cycle. In terms of treatment, standalone biomedical or psychological interventions have limited efficacy, necessitating an interdisciplinary and integrated approach. Pharmacological treatment requires attention to drug interactions between antidepressants and breast cancer medications such as tamoxifen. Psychological interventions, including cognitive-behavioral therapy, mindfulness-based yoga, and virtual reality technology, can effectively alleviate depressive symptoms and improve treatment adherence. Additionally, emerging approaches such as traditional Chinese medicine, neuromodulation techniques, and gut microbiota regulation show considerable potential for intervention. These findings provide new insights for understanding and clinically managing the comorbidity of depression and breast cancer.

## 1 Introduction

Depression is a common mental disorder characterized by significant and persistent low mood, loss of interest, and lack of energy as its core features. It is often accompanied by changes in sleep and appetite, as well as impaired cognitive function. In severe cases, individuals may experience suicidal thoughts or behaviors, causing serious harm to psychological functioning, social interaction, and quality of life (1). According to estimates from the World Health Organization (WHO), approximately 350 million people worldwide suffer from depression, and the prevalence is increasing year by year, making it a major contributor to the global burden of disease (2) Meanwhile, breast cancer is the most common cancer among women and a leading cause of cancer-related deaths. It causes up to 680,000 female deaths annually, accounting for 11.7% of all new cancer cases and 6.9% of new cancer-related deaths worldwide (3–5).

In recent years, extensive clinical and basic research has confirmed a close and complex bidirectional relationship between depression and breast cancer. This association is manifested not only in the fact that the diagnosis and treatment of breast cancer can induce or exacerbate depressive symptoms, but studies are increasingly revealing that depression itself is an independent risk factor for the development and progression of breast cancer (6). The two conditions interact, potentially a vicious cycle that may be collectively linked to poorer clinical outcomes. Relevant data indicate that the prevalence of depression in the general population is approximately 6.4%, whereas among breast cancer patients, this rate can be as high as 32.2% (7). This comorbid state has profound adverse effects on the disease progression and clinical outcomes of patients. These patients often experience more severe cancer-related pain, more extreme cancer-related fatigue, a significantly reduced overall quality of life, and it can even impact survival rates.

Given the high comorbidity rate of depression and breast cancer and their mutually exacerbating vicious cycle, traditional single−discipline diagnostic and therapeutic models are inadequate in addressing such complex comorbidities. The fragmented management by psychology and oncology often leads to insufficient assessment of the patient’s overall condition and a lack of coordinated treatment strategies, making it difficult to simultaneously address mental health and tumor control. Moreover, neglecting psychosocial factors may adversely affect treatment adherence and physical recovery. Furthermore, the interplay between these two conditions at the pathophysiological level (such as neuroendocrine dysregulation and immune−inflammatory alterations) further underscores the limitations of relying solely on a single disciplinary perspective. Therefore, this article aims to comprehensively review the association between depression and breast cancer, its underlying pathophysiological mechanisms, and clinical management strategies from an interdisciplinary and integrated perspective. This approach seeks to provide new insights for deepening the understanding of the nature of this comorbidity and for developing multimodal comprehensive intervention strategies.

## 2 Search strategy

The literature search was primarily conducted in the PubMed and Web of Science databases, covering the period from database inception to April 2026. The English search strategy combined Medical Subject Headings (MeSH) with free-text terms. The main search query was structured as follows: (“Depressive Disorder” OR “Depression”) AND (“Breast Neoplasms” OR “Breast Cancer”) AND (“Mechanism” OR “Pathophysiology” OR “Treatment” OR “Comorbidity”).

The literature screening process was performed as follows: First, two investigators independently screened titles and abstracts to exclude irrelevant studies (e.g., non-clinical research and case reports). Subsequently, full texts were retrieved and reviewed to focus on original articles and high-quality reviews that explored the bidirectional association between depression and breast cancer, neuroendocrine mechanisms, immune-inflammatory pathways, the gut-brain axis, and clinical interventions. Additionally, manual searches of reference lists from key articles were performed to identify any missed studies. Finally, after duplicate removal, screening, and quality assessment, the remaining eligible studies were included in this review.

## 3 The association between depression and breast cancer

### 3.1 Behavioral links between depression and breast cancer

The characteristic persistent low mood and negative-pessimistic state of depression serve as significant psychological stressors impacting physical health. These factors initiate a series of physiological and behavioral changes through the following pathways, thereby establishing a risk bridge for breast diseases (8): ① Stress-related negative emotions directly lead to significant psychological distress and increase the risk of mental disorders, adversely affecting mental health; ② Negative emotions trigger biological dysfunction, manifesting as neuroendocrine and immune system disturbances, along with abnormal elevations in various clinical indicators; ③ Individuals coping with negative emotions often tend to adopt risky health behaviors such as smoking, excessive alcohol consumption, or poor diet as buffering strategies; ④ Depressive states significantly weaken patients’ willingness and behavior to actively seek and adhere to healthcare, thereby delaying early diagnosis and intervention. Studies have confirmed (9) that unhealthy lifestyle plays a central mediating role in the above multiple pathways. It is precisely by fostering these behaviors that negative emotions significantly increase the incidence of breast cancer. Depression increases the risk of breast cancer onset through multiple pathways, including psychological distress, neuroendocrine dysregulation, unhealthy lifestyles, and healthcare avoidance behaviors. These findings suggest that depression is not merely a psychological complication of breast cancer, but rather a significant upstream risk factor driving its pathogenesis.

### 3.2 Diagnostic links between depression and breast cancer

The psychological impact on patients upon receiving a breast cancer diagnosis is substantial, with over 20% developing a depressive state that meets diagnostic criteria following confirmation of their diagnosis (10). Although some patients may have a degree of psychological preparedness, the sudden nature of the confirmed diagnosis can still exceed an individual’s coping capacity, leading to acute stress reactions. A study indicated (11) that approximately 30% of depressed patients exhibit symptom clusters similar to post-traumatic stress disorder (PTSD), including re-experiencing the diagnostic scenario, avoiding healthcare-related topics, and hypervigilance. In other words, a breast cancer diagnosis constitutes a major psychological stressor, and its intricate association with depressive symptoms persists across the entire disease trajectory—from initial suspicion to long-term survivorship.

### 3.3 The link between depression and breast cancer prognosis

The co-occurrence of depression and breast cancer worsens patient prognosis through multiple physiological, psychological, and behavioral pathways, creating a self-reinforcing vicious cycle. Depression-induced neuroendocrine dysregulation (e.g., persistent HPA axis activation) and an immunosuppressed state (e.g., reduced NK cell activity, elevated pro-inflammatory cytokines) act synergistically with breast disease progression. Such alterations in the immune microenvironment may enhance tumor invasiveness and metastatic potential (12–14). Furthermore, one study indicated that breast cancer patients with a history of chronic psychological stress not only had a significantly higher body mass index but also a markedly increased incidence of aggressive subtypes, such as HER-2 overexpression (15). Another study demonstrated that depression is independently associated with increased mortality in breast cancer patients, and comorbidity further elevates this risk (16). This suggests that psychological stress may influence tumor biological behavior by modulating metabolic indicators and the immune microenvironment. On the behavioral level, depressive symptoms (e.g., hopelessness, lack of energy) directly impair treatment adherence, leading to chemotherapy interruptions, missed medications, and other behaviors that compromise therapeutic efficacy.

## 4 Mechanisms by which depression contributes to breast cancer

### 4.1 Neuroendocrine dysregulation

#### 4.1.1 HPA axis hyperactivation

The hypothalamic-pituitary-adrenal (HPA) axis is a core component of the human neuroendocrine system and plays a vital role in maintaining stress response, metabolism, immune function, and biological rhythms. In the pathophysiology of depression, the negative emotional state itself acts as a chronic psychological stressor, leading to persistent activation and subsequent hyperactivation of the HPA axis, creating a vicious cycle (17). This activation begins in the paraventricular nucleus of the hypothalamus, where neurons synthesize and release corticotropin-releasing hormone (CRH) into the pituitary portal system. CRH then acts on the anterior pituitary to stimulate the secretion of adrenocorticotropic hormone (ACTH), ultimately triggering the adrenal cortex to produce glucocorticoids (GCs). This sequential activation from the central nervous system to the periphery results in chronically elevated levels of GCs in depressed patients, a hallmark feature of HPA axis hyperactivation.

Research indicates (18) that the physiological effects of GCs and the precision of their negative feedback regulation depend critically on their binding to two receptor types in the brain: the mineralocorticoid receptor (MR) and the glucocorticoid receptor (GR). GCs have a very high affinity for MR, which is predominantly located in limbic brain regions such as the hippocampus, areas crucial for memory and emotion regulation. In contrast, GR has a lower affinity for GCs and is widely expressed throughout the brain, including the hypothalamus and pituitary. Under baseline conditions, GCs primarily bind to MR to maintain normal HPA axis tone. However, when stress significantly elevates GC concentrations, GR becomes substantially activated, triggering a negative feedback mechanism that inhibits further secretion of CRH and ACTH, thereby restoring HPA axis activity to normal levels. Another study shows (19) that impaired GR function is a central factor in HPA axis dysregulation. Potential mechanisms include downregulation of GR expression or abnormalities in GR signal transduction pathways. When GR function is compromised, the negative feedback inhibition mediated by GCs in key brain regions such as the pituitary, hypothalamus, and hippocampus is significantly weakened, even in the presence of persistently high GC levels. This results in a failure to effectively suppress the HPA axis, leading to continued oversecretion of CRH and ACTH and prolonged stimulation of the adrenal cortex, thereby sustaining the high GC state. This impairment in GR-mediated negative feedback not only exacerbates HPA axis hyperactivation but may also be a key biological substrate for clinical manifestations in depressed patients, such as cognitive dysfunction and emotional dysregulation. In summary, the resulting excessive GCs act as a “trigger point” for pathophysiological changes in depressed individuals. By influencing multiple downstream mechanisms, they alter the microenvironment of breast tissue and significantly increase the risk of breast disease.

#### 4.1.2 HPO axis dysregulation

The breast, as a classic hormone-target organ, undergoes development, involution, and cyclical changes that are highly dependent on the hormonal homeostasis regulated by the hypothalamic-pituitary-ovarian (HPO) axis. The pulsatile secretion of gonadotropin-releasing hormone (GnRH) acts as the critical “switch” initiating the entire HPO axis function. Excessively high concentrations of glucocorticoids (GCs) directly act on hypothalamic GnRH neurons, suppressing the activity of their pulse generator and disrupting the rhythm and amplitude of GnRH secretion (20). Furthermore, corticotropin-releasing hormone (CRH) potently inhibits GnRH release and can also stimulate the hypothalamus to increase the production of endogenous opioid peptides, such as endorphins, which are known potent inhibitors of GnRH secretion (21, 22). Even if small amounts of GnRH reach the pituitary, high GC levels reduce the sensitivity of the anterior pituitary to GnRH (23). This results in a diminished pituitary response to secrete follicle-stimulating hormone (FSH) and luteinizing hormone (LH).

**Figure 1:**
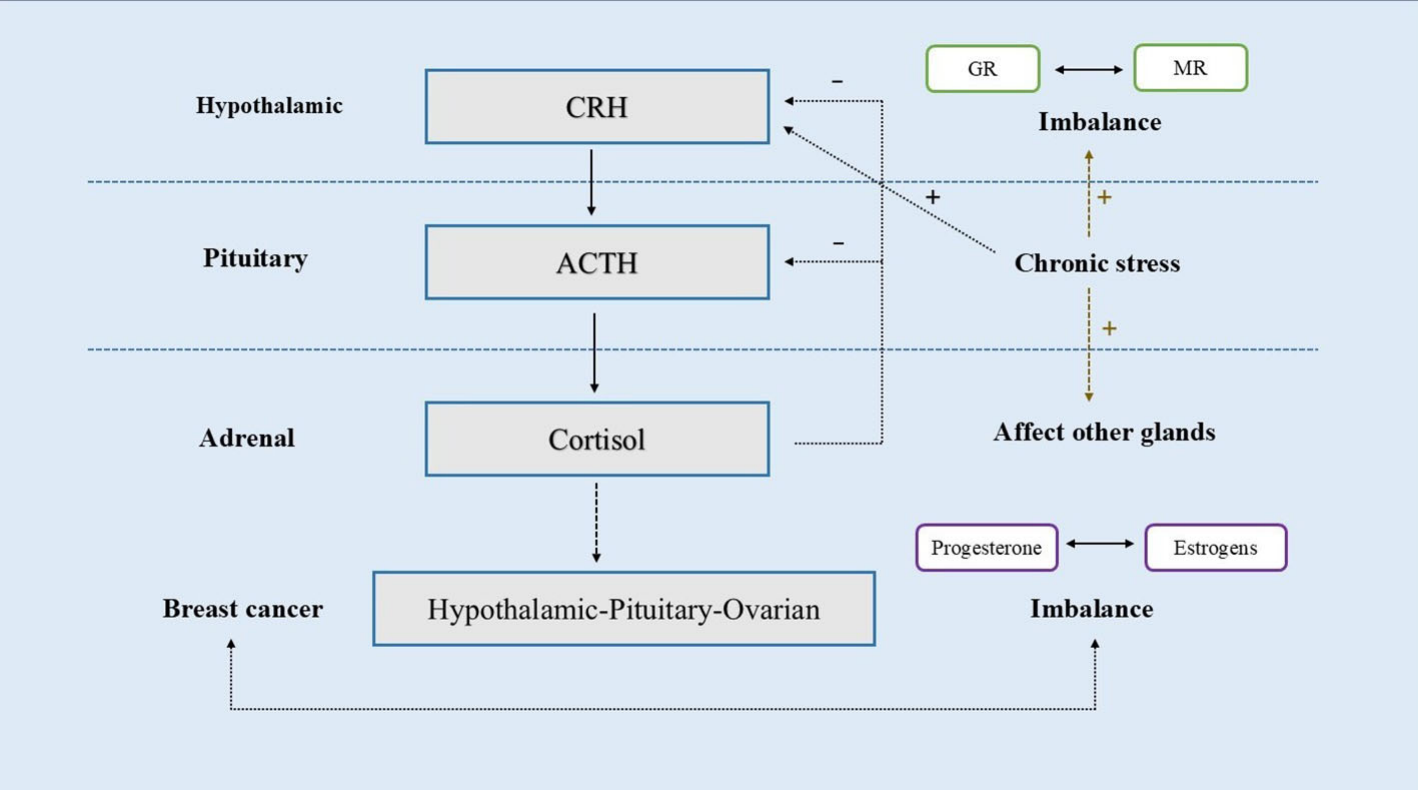
Negative emotions such as depression trigger the hyperactivity of the HPA axis. Moreover, the imbalance between glucocorticoid receptors and mineralocorticoid receptors mediates the impairment of the pituitary negative feedback mechanism, leading to an increase in the levels of hormones such as cortisol. This, in turn, causes the disorder of the HPO axis and induces the occurrence and development of breast diseases.

This dysregulation often leads to two key consequences: first, anovulation or luteal phase deficiency, causing insufficient progesterone secretion; and second, the emergence of a relative or absolute estrogen dominance. Estrogen is a potent promoter of breast epithelial cell proliferation, and its persistent, excessive stimulation is a central driver of breast tissue hyperplasia and even carcinogenesis (24). Conversely, the relative deficiency of progesterone weakens its antagonistic effect against estrogen-driven proliferation and its role in inducing differentiation (25). This state of “estrogen dominance” disrupts the normal balance between breast cell proliferation and apoptosis, significantly increasing the risk of hyperplastic breast diseases and breast cancer. The disruption of sex hormones caused by HPA axis hyperactivation is illustrated in **Figure 1**.

#### 4.1.3 Prolactin dysregulation

Prolactin (PRL), a polypeptide hormone secreted by the anterior pituitary, promotes mammary gland development and lactation, and is also closely associated with stress resilience and mood regulation (26). Its secretion is primarily under tonic inhibition by dopamine released from the hypothalamus (27). Chronic stress and depression can suppress the activity of tuberoinfundibular dopaminergic neurons in the hypothalamus, reducing dopamine synthesis and release. This attenuates the inhibitory control over pituitary lactotrophs, leading to increased PRL secretion—even though PRL itself can exert an inhibitory influence on HPA axis reactivity (28).

One study (29), utilizing transgenic NRL-PRL models, demonstrated that PRL can independently increase mammary stem cells, regulate transcriptional programs involved in differentiation, and enhance stem cell activity, irrespective of ovarian steroids (estrogen/progesterone). Furthermore, PRL was shown to act synergistically with estrogen and progesterone to enhance Wnt signaling-associated stem cell functions, highlighting its critical role in mammary pathogenesis by influencing the epithelial hierarchy. Another study indicated (30) that PRL activates the serine/threonine kinase NEK3, which plays an important role in enhancing the migration and invasion of breast cancer cells. Moreover, PRL can induce tyrosine phosphorylation of the serine/threonine kinase PAK1 and recruit the tyrosine phosphatase PTP-PEST to inactivate focal adhesion kinase (FAK). This process enhances focal adhesion turnover dynamics, thereby driving the migration and metastasis of breast cancer cells (31).

#### 4.1.4 Insulin resistance

Insulin resistance is a significant aspect of the endocrine disturbances associated with depression. The elevated glucocorticoid (GC) levels resulting from HPA axis hyperactivation interfere with normal insulin signal transduction, markedly reducing insulin sensitivity in peripheral tissues (such as muscle and adipose tissue) and inducing insulin resistance (32, 33). Insulin resistance leads to compensatory hyperinsulinemia and dysregulated glucose metabolism. This metabolic abnormality further contributes to lipid metabolism disorders and promotes the redistribution of body fat, particularly leading to ectopic accumulation of visceral fat (abdominal fat) (34). It is crucial to emphasize that adipose tissue, especially dysfunctional visceral fat, is not merely a passive energy storage organ but an active endocrine organ. The various adipokines it secretes play a key role in the metabolic and hormonal imbalances related to depression.

**Figure 2:**
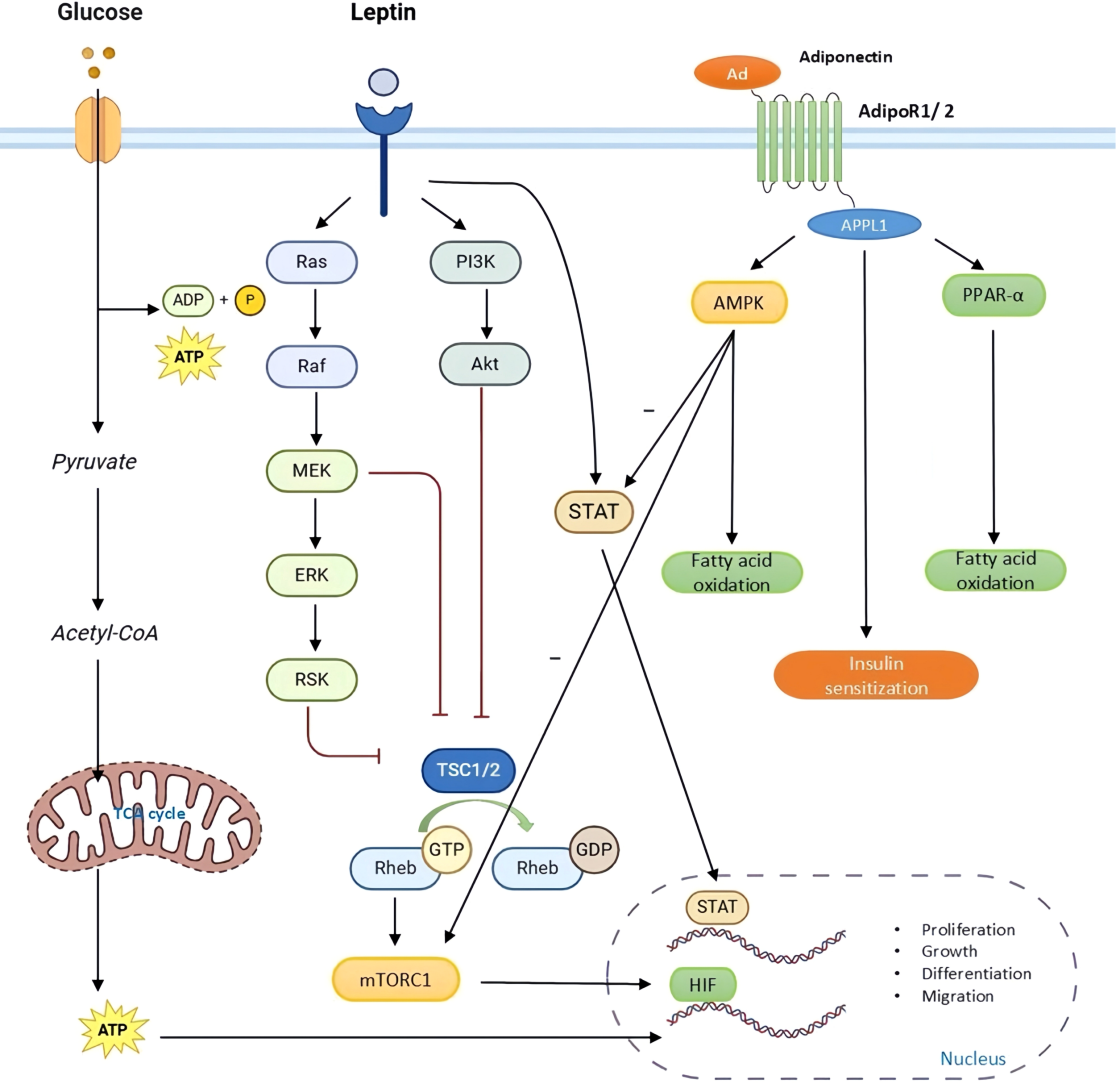
Leptin promotes the proliferation, growth, differentiation, and migration of tumor cells through the MEK-ERK, PI3K-Akt, and STAT pathways. Adiponectin improves lipid metabolism disorders and increases insulin sensitivity through pathways such as AMPK and PPAR, and counteracts the signaling pathways of leptin, thus affecting metabolism.

Adipose tissue expansion leads to an adverse shift in the adipokine secretion profile, characterized by elevated leptin levels and a significant reduction in the secretion of adiponectin, which has beneficial metabolic effects (35). Research indicates (36) that leptin, acting as a pro-proliferative factor, can directly promote the proliferation, growth, differentiation, and migration of mammary epithelial cells and even breast cancer cells by activating signaling pathways such as MAPK/ERK, PI3K/Akt, and JAK/STAT. Another study suggests (37) that adiponectin plays an important antagonistic and protective role. By activating AMPK and PPAR-α pathways, it not only improves lipid metabolism and insulin sensitivity but also directly inhibits leptin-mediated pro-proliferative signaling within breast tissue. Consequently, the “high leptin/low adiponectin” state induced by depression deprives breast tissue of an important intrinsic protective mechanism. See **Figure 2**.

In summary, chronic HPA axis hyperactivity serves as the initiating event that disrupts the HPO axis, prolactin secretion, and insulin sensitivity in a cascading manner, thereby establishing a tumorigenic endocrine microenvironment characterized by sustained elevations in glucocorticoids, estrogens, and prolactin. These findings indicate that the systemic dysregulation of the neuroendocrine system acts as the core physiological bridge linking psychological stress to breast carcinogenesis.

### 4.2 Suppression of immune cells

The immune system performs an immune surveillance function, capable of identifying and eliminating abnormal mutated cells within the body, thereby maintaining internal homeostasis. However, the overall immune capacity is suppressed in patients with depression (38), rendering the body more susceptible to disease.

T cells are the core of adaptive immunity. A study using a mouse model transplanted with 4T1 breast cancer cells found that chronic restraint stress significantly promoted tumor growth, and the number of CD8+ T cells was markedly reduced in the peripheral blood, spleen, and tumor-infiltrating lymphocytes of stressed mice, indicating impaired anti-tumor immune responses (39). Glucocorticoids (GCs) primarily suppress the initial activation and clonal proliferation of T cells through GR-mediated genomic effects, which include downregulating the expression of the T-cell receptor (TCR) and co-stimulatory molecules like CD28, and interfering with TCR signal transduction and IL-2 production (40). Research shows that GCs can induce apoptosis in T cells at various developmental stages; not only are immature CD4+CD8+ double-positive thymocytes in the central immune system particularly sensitive to GCs, but activated effector T cells in the periphery are also susceptible to GC-induced apoptosis (41). Furthermore, GCs drive an imbalance in CD4+ T cell subsets, skewing the immune response towards a Th2 profile while suppressing the differentiation of Th1 and Th17 cells and the secretion of their associated cytokines (e.g., IFN-γ, IL-17), thereby weakening the cell-mediated immunity crucial for anti-tumor defense (42). Notably, GCs are also closely associated with an increased frequency of regulatory T cells (Tregs) in the circulation and/or inflammatory tissues, potentially by inducing the upregulation of the transcription factor FoxP3, which mediates Treg generation and function, further exacerbating the immunosuppressive state within the tumor microenvironment (43, 44).

B cells play an irreplaceable role in humoral immunity. One study (45) established a mouse model of triple-negative breast cancer (TNBC) and depression comorbidity using chronic restraint stress (CRS) and 4T1 tumor transplantation. It found an increased B cell count, decreased CD4+ and CD8+ T cell counts (with no statistically significant difference in Treg count), and observed similar results even in the context of PD-L1 inhibitor therapy. This suggests that abnormal B cell expansion in the depressive tumor microenvironment might be related to the suppressed state of T cells and may not be readily reversed by immune checkpoint blockade. Another study (46) on 101 hip fracture patients and 43 healthy age-matched controls 6 weeks post-injury found that 38 hip fracture patients (37%) developed depressive symptoms. In these depressed patients, a significantly lower frequency of regulatory B cells (Bregs) and a marked decrease in IL-10 production by Bregs were observed. These findings suggest that, under certain pathological conditions, depression may impair the immunosuppressive function mediated by Bregs. Although this has not yet been confirmed in breast cancer, it raises the possibility that similar Breg dysfunction may occur in breast cancer patients with comorbid depression, potentially affecting tumor immunity and overall disease progression. It is important to note that most existing research is based on peripheral blood measurements, and variations in sample size, detection methods, and patient populations exist across studies. Therefore, the specific mechanisms and clinical significance of B cell changes require further validation. Future research might utilize more precise single-cell technologies or functional experiments to explore the role of B cells in the pathogenesis and treatment of depression.

Natural Killer (NK) cells are the first line of defense in tumor immune surveillance. Their cytotoxic activity relies on the synthesis and release of cytotoxic granules like perforin and granzymes. Perforin creates pores in the target cell membrane, allowing granzymes (e.g., Granzyme B) to enter the cell and activate caspase cascades, inducing apoptosis (47). GCs directly impair NK cell killing capacity by inhibiting the transcription and synthesis of key cytotoxic granules like granzyme A (48). A study using a C57BL/6 mouse model found that dexamethasone treatment reduced the number of splenic NK cells and downregulated the expression of their surface activating receptors (e.g., NKG2D), which are crucial for recognizing stress-induced ligands (like MICA/B) on tumor cells, thereby impairing their ability to identify and kill tumor cells (49).

Dendritic cells (DCs) are the most potent professional antigen-presenting cells, essential for initiating T cell-specific immunity. Studies show that GCs can inhibit the expression of MHC class II molecules and co-stimulatory molecules like CD86 on DCs, keeping them in an immature or semi-mature state and thus unable to effectively present antigens and activate naïve T cells (50). Secondly, GCs can induce DCs to adopt a regulatory, tolerogenic phenotype, which in turn promotes the generation of regulatory T cells, ultimately inducing immune tolerance (51).

Macrophages have a dual role in the tumor microenvironment. GCs can promote the polarization of macrophages towards the M2 phenotype. M2 macrophages highly express arginase, promote tissue repair and angiogenesis, and secrete inhibitory cytokines like IL-10 and TGF-β, creating a microenvironment favorable for tumor growth and metastasis (52).

In summary, GCs synergistically suppress the killer functions of T cells and NK cells, impair the antigen-presenting capacity of DCs, and promote macrophage polarization towards the M2 phenotype, collectively shaping a highly immunosuppressive tumor microenvironment. This multi-targeted immunosuppressive effect significantly weakens the body’s ability to conduct immune surveillance and clear tumor cells, thereby creating favorable conditions for the initiation and progression of breast cancer.

### 4.3 Dysregulation of inflammatory cytokine levels

Cytokines are small proteins produced by immune cells (such as monocytes/macrophages, T cells) and some non-immune cells (such as endothelial cells) in response to stimulation. Functionally, they are categorized into pro-inflammatory cytokines (e.g., IL-1β, IL-6, TNF-α) and anti-inflammatory cytokines (e.g., IL-10, TGF-β). Under physiological conditions, the dynamic balance between these two types of cytokines is fundamental for maintaining immune homeostasis. However, substantial clinical evidence indicates that patients with depression exhibit a clear state of low-grade systemic inflammation, characterized by persistently elevated levels of pro-inflammatory cytokines in the peripheral blood and cerebrospinal fluid, leading to an imbalance between pro-inflammatory and anti-inflammatory cytokine levels.

Research shows that under the influence of inflammatory factors, the integrity of the blood-brain barrier (BBB) is compromised, allowing peripheral inflammatory cytokines to translocate into the central nervous system (53). Another study indicates that cytokines can also transmit “sickness signals” to the brain via pathways such as vagal nerve afferent signals or by acting on brain regions lacking a complete BBB, thereby strongly activating microglia, the brain’s resident immune cells (54). The sustained activation of microglia is a key amplifying step in the neuroinflammatory circuitry of depression. Activated microglia tend to polarize towards a pro-inflammatory M1 phenotype, subsequently releasing more pro-inflammatory cytokines and neurotoxic substances, forming a positive feedback loop that exacerbates neuroinflammation (55). This chronic inflammatory microenvironment disrupts neural function at multiple levels: First, it interferes with the metabolism of monoamine neurotransmitters by activating indoleamine 2,3-dioxygenase (IDO), shunting tryptophan metabolism towards the production of neurotoxic metabolites while reducing the synthesis of serotonin (5-HT) (56). Second, it impairs neuroplasticity; inflammatory cytokines can inhibit astrocyte function, leading to downregulated expression of brain-derived neurotrophic factor (BDNF), which in turn causes neuronal atrophy and reduced synaptic connectivity in brain regions such as the hippocampus and prefrontal cortex (57). Third, it contributes to HPA axis dysfunction; inflammatory signals not only directly stimulate the HPA axis causing excessive GC secretion but can also induce glucocorticoid receptor resistance, disrupting normal negative feedback and keeping the body in a prolonged state of stress (58).

**Figure 3:**
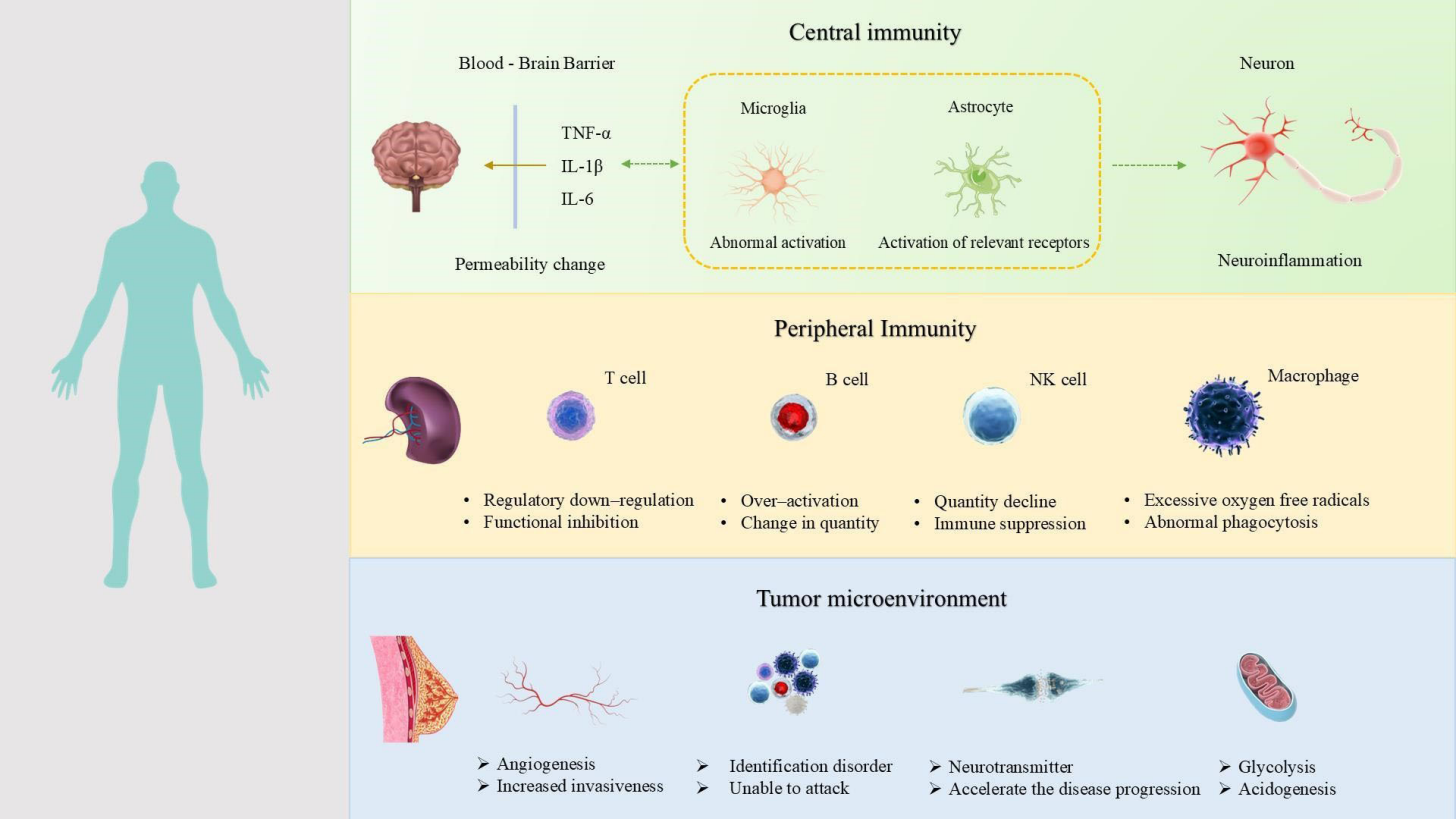
This clearly illustrates the cascade of mechanisms by which depression promotes the development of mammary tumors: chronic stress initially disrupts the homeostasis of central immunity by increasing blood-brain barrier permeability, allowing pro-inflammatory factors (e.g., TNF-α, IL-6) to infiltrate the central nervous system, triggering neuroinflammation and resulting in neuronal damage. This, in turn, drives comprehensive dysregulation of peripheral immunity, where the functions of key immune cell (such as T cell, B cell, NK cell, and macrophage) are suppressed or disrupted, leading to a failure of immune surveillance. Ultimately, this systemic immunosuppression shapes a pro-tumor microenvironment locally, characterized by angiogenesis, immune evasion, metabolic reprogramming, and neurotransmitter-accelerated disease progression, collectively creating the necessary conditions for tumor initiation and advancement.

The release of excessive pro-inflammatory cytokines induces the production of large amounts of reactive oxygen species (ROS) and reactive nitrogen species (RNS), which directly damage cellular DNA and can even lead to mutations in BRCA1/2 genes, potentially initiating breast cancer development (59). Studies show that cytokines like IL-6 and TNF-α can stimulate the generation of new blood vessels around tumor tissue (angiogenesis), providing a crucial pathway for further tumor growth and metastasis (60). Another study indicates that inflammatory cytokines can influence the metabolic pathways of estrogen in the body; they can promote the activity of the enzyme aromatase, thereby increasing estrogen synthesis (61). Higher local estrogen levels in breast tissue continuously promote the proliferation of estrogen receptor-positive breast cells, significantly increasing the risk of carcinogenesis. Changes in the function and levels of cytokines in patients with depression are summarized in **Table 1**. The immune mechanisms linking depression and breast cancer are illustrated in **Figure 3**.

**Table 1:** alterations in cytokine function and levels in patients with depression.

Cytokine	Function	Type	Level	Reference
IL-1β	Induce fever, T cell activation, and macrophage activation	Proinflammatory factor	Increase	(62)
IL-2	Induces T-cell and B-cell activation and enhances antigen presentation.	Proinflammatory factor	Increase	(63)
Interleukin-3 (IL-3)	Stimulate the proliferation of hematopoietic stem cells and enhance the phagocytic function of macrophages	Proinflammatory factor	Increase	(64)
Interleukin-4 (IL-4)	Induce B cell activation	Anti-inflammatory cytokine	Decrease	(65)
Interleukin-5 (IL-5)	Promote the growth and differentiation of eosinophils	Anti-inflammatory cytokine	Increase	(66)
IL-6	Induce T and B cell growth and differentiation, produce acute phase proteins, and trigger fever	Proinflammatory factor	Increase	(67)
Interleukin-8 (IL-8)	Attract and activate neutrophils	Proinflammatory factor	Increase	(68)
Interleukin-9 (IL-9)	Induce mast cell activity and stimulate Th activity	Proinflammatory factor	Increase	(69)
IL-10	Strongly inhibit macrophage function and induce anti-inflammatory behaviors	Anti-inflammatory cytokine	Decrease	(64)
Interleukin-12 (IL-12)	Activate NK cells and induce CD4 T cell differentiation	Proinflammatory factor	Increase	(70)
Interleukin-13 (IL-13)	Inhibit macrophage inflammatory cytokine production	Anti-inflammatory cytokine	Increase	(71)
Interleukin-17A (IL-17A)	Induce epithelial cells, endothelial cells, astrocytes, and fibroblasts to produce cytokines and promote inflammation	Proinflammatory factor	Increase	(64)
Interleukin-18 (IL-18)	Induce T cells and NK cells to produce IFN-γ	Proinflammatory factor	Increase	(72)
IFN-γ	Promote the secretion of related cytokines and increase the expression of major histocompatibility complex Class II molecules	Proinflammatory factor	Decrease	(73)
CC Chemokine ligand 2 (CCL2)	Attract immune cells to the site of inflammation: T cells, monocytes, etc	Proinflammatory factor	Increase	(74)
CC Chemokine ligand 3 (CCL3)	Attract immune cells to the site of inflammation: T cells, monocytes, fibroblasts, osteoclasts, etc	Proinflammatory factor	Increase	(75)
CC Chemokine ligand 4 (CCL4)	Recruits immune cells to inflammatory sites and modulates immune cell function.	Proinflammatory factor	Increase	(76)
CC Chemokine ligand 5 (CCL5)	Target T cells, NK cells, eosinophils, neutrophils, etc	Proinflammatory factor	Increase	(77)
CC Chemokine ligand 7 (CCL7)	Attract and activate T cells and promote the aggregation of immune cells	Proinflammatory factor	Increase	(74)
CC Chemokine ligand 8 (CCL8)	Regulate the migration and activation of neutrophils	Proinflammatory factor	Increase	(74)
CC Chemokine ligand 11 (CCL11)	Recruit eosinophils associated with anaphylaxis	Proinflammatory factor	Increase	(64)
TNF-β	Promote inflammation and endothelial activation	Proinflammatory factor	Increase	(78)
Transforming Growth Factor-β (TGF-β)	Exhibit anti-inflammatory properties, counteracting the effects of IL-6	Anti-inflammatory cytokine	Decrease	(64)

In summary, the persistent low-grade inflammatory state in depressed patients not only precipitates neuroinflammation by disrupting the blood-brain barrier but also promotes angiogenesis, estrogen synthesis, and DNA damage via peripheral pro-inflammatory factors, thereby inflicting bidirectional “brain-body” damage. These findings indicate that inflammatory cytokines serve as critical mediators in the crosstalk between psychological stress and tumor biological behavior.

### 4.4 Imbalance in tryptophan metabolic pathways

Tryptophan, an essential amino acid, is a crucial precursor for synthesizing serotonin (5-HT) and melatonin, playing a vital role in regulating mood, sleep, and circadian rhythms. The kynurenine pathway (KP) generates neuroactive metabolites that significantly impact neuroimmune balance. The dynamic equilibrium between these two metabolic pathways is central to maintaining neuropsychiatric function and immune homeostasis, and their dysregulation is closely linked to various psychiatric and immune-related diseases.

Chronic stress and inflammation lead to the sustained upregulation of the rate-limiting enzymes in the KP—indoleamine 2,3-dioxygenase 1 (IDO1) and tryptophan 2,3-dioxygenase 2 (TDO2). Furthermore, elevated levels of pro-inflammatory cytokines (such as IL-1β and IL-6) enhance this effect (56). This metabolic imbalance shunts tryptophan away from 5-HT synthesis towards the production of kynurenine and its downstream metabolites. The resultant deficiency in 5-HT is central to the symptoms of depression. Within the central nervous system, an increased ratio of the neurotoxic metabolite quinolinic acid (QA) to the neuroprotective metabolite kynurenic acid (KYNA) promotes excitotoxicity and oxidative stress, leading to the structural and functional neuronal damage observed in depression (79).

**Figure 4:**
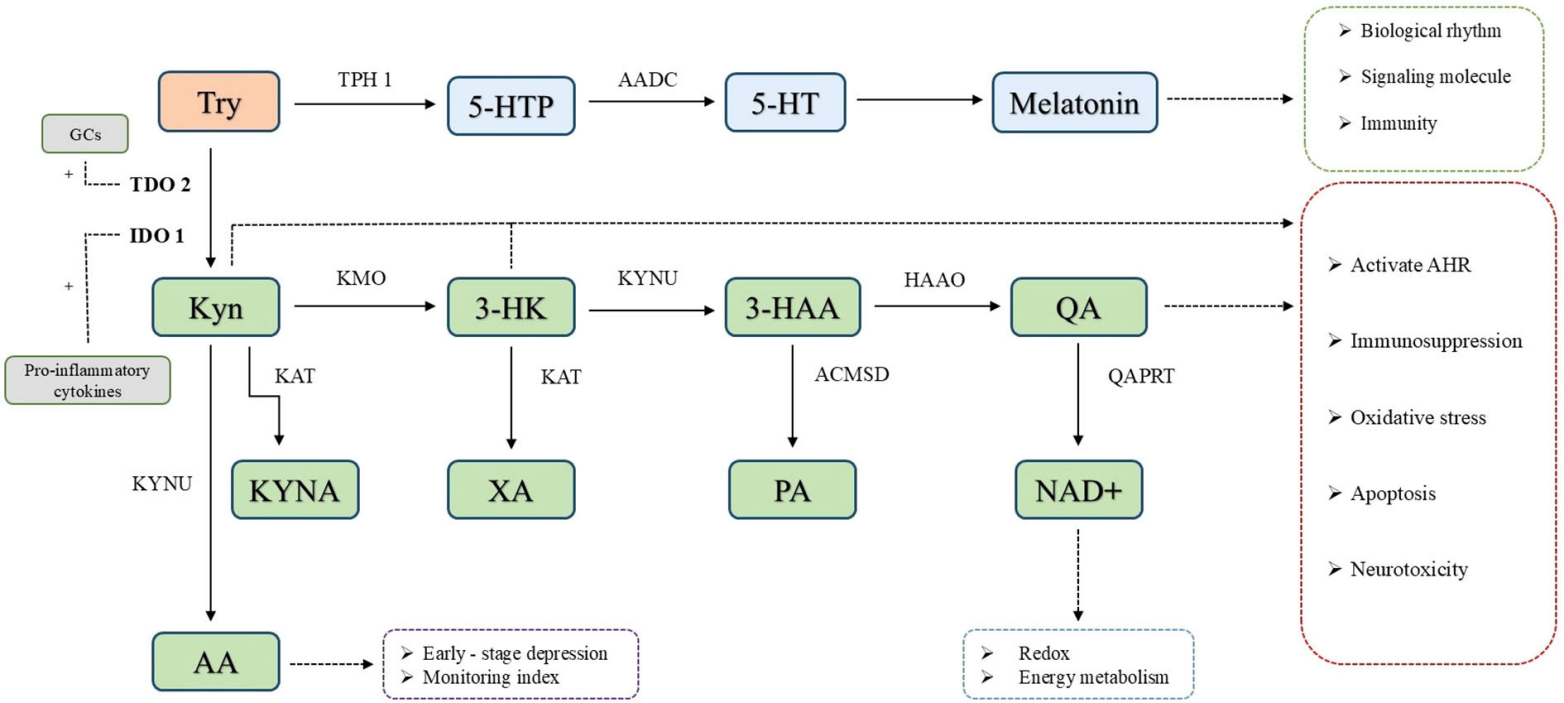
Tryptophan metabolism primarily proceeds through two major pathways: the upstream serotonin pathway, where TPH1 and AADC catalyze the production of the neurotransmitter 5-HT and melatonin, regulating biological rhythms; and the downstream kynurenine pathway, initiated by IDO1/2 or TDO, which, through enzymes such as KMO, generates metabolites with neurotoxic (e.g., QA) or neuroprotective (e.g., KYNA) properties, ultimately leading to the synthesis of NAD +. Signals such as cortisol and pro-inflammatory cytokines influence pathway balance by regulating the expression of key enzymes, and their dysregulation is closely associated with pathological processes including immunosuppression and early-stage depression. 5-HTP, 5-hydroxyindoleacetic acid; 5-HT, 5-hydroxytryptamine; Kyn, Kynurenine; 3-HK, 3-hydroxykynurenine; AA, anthranilic acid; KYNA, kynurenic acid; XA, xanthurenic acid; 3-HAA, 3-hydroxyanthranilic acid; QA, quinolinic acid; PA, picolinic acid; NAD+, Nicotinamide Adenine Dinucleotide.

Concurrently, in the peripheral tumor microenvironment, kynurenine and its derivatives can activate the aryl hydrocarbon receptor (AhR). This receptor modulates the differentiation of regulatory T cells (Tregs) and directly suppresses cytotoxic T cell function, thereby fostering an immunosuppressive microenvironment conducive to breast tumor growth and metastasis (80). The tryptophan metabolic pathways and their functions are illustrated in **Figure 4**.

In summary, stress-induced tryptophan metabolic reprogramming leads to both central neurotransmitter (5-HT) depletion and the formation of a peripheral immunosuppressive microenvironment, thereby tightly linking neurobiochemical alterations with tumor immune evasion. This highlights tryptophan metabolic imbalance as a critical nexus connecting neurochemistry and immune metabolism in the comorbidity mechanism of depression and breast cancer.

### 4.5 Gut microbiota dysbiosis

The gut-brain axis represents a complex bidirectional communication network linking the gastrointestinal tract and the central nervous system. Psychological stress and negative emotional signals from the brain can directly disrupt gut homeostasis, leading to microbial dysbiosis. This dysbiosis is characterized by a reduction in beneficial bacteria (e.g., Lactobacillus, Bifidobacterium) and an expansion of pro-inflammatory bacterial populations (e.g., lipopolysaccharide (LPS)-producing Gram-negative bacteria), an imbalance positively correlated with the severity of depression (81). These altered microbes can, in turn, influence brain function and behavior by producing neuroactive substances (e.g., serotonin, GABA) and triggering systemic inflammatory responses. Research indicates (82) that depression is closely associated with impaired intestinal barrier integrity, manifested by the downregulation of tight junction proteins (such as claudin, occludin, and tricellulin). This pathological “leaky gut” allows bacterial pathogen-associated molecular patterns (e.g., LPS) to translocate into the systemic circulation. There, they bind to Toll-like receptor 4 (TLR4) on immune cells (e.g., monocytes, macrophages), triggering a robust pro-inflammatory cascade involving the activation of key signaling pathways like NF-κB and MAPK. This drives a systemic low-grade inflammatory state, contributing to a cytokine storm in the serum and cerebrospinal fluid (82, 83).

Furthermore, another study (84) demonstrated that depression-associated dysbiosis of the gut microbiota involved in the enterohepatic circulation can significantly upregulate the activity of bacterial β-glucuronidase. Under normal physiological conditions, the liver conjugates estrogens with glucuronic acid to form water-soluble estrogen–glucuronide conjugates, which are excreted via bile into the intestine and ultimately eliminated from the body. However, in the context of depression, heightened β-glucuronidase activity within the gut cleaves the glucuronic acid moiety from these conjugated estrogens, regenerating free, biologically active estrogen molecules. These deconjugated estrogens are then reabsorbed across the intestinal epithelium into the portal circulation, leading to elevated systemic bioavailability of estradiol and other estrogenic hormones. Sustained increases in circulating estrogen levels, driven by this microbiota–enzyme axis, can promote the proliferation of estrogen receptor-positive mammary epithelial cells, upregulate the expression of estrogen-responsive oncogenes, and create a hormonal microenvironment conducive to the initiation and progression of breast cancer, potentially also contributing to resistance to endocrine therapy.

In summary, bidirectional dysregulation of the gut-brain axis increases circulating estrogen reabsorption through the “microbiome-gut-liver axis” and triggers systemic inflammation via “leaky gut”, thereby synergistically promoting both mammary tumor growth and depressive behaviors, thus identifying novel targets for comorbidity intervention.

## 5 Management of comorbid depression and breast cancer

Although the treatment approaches for depression and breast cancer individually are relatively well-established, managing their co-occurrence is not straightforward and presents a unique set of challenges and complexities. Particularly, as the 5-year survival rate for breast cancer patients now exceeds 80%, focusing on the physical functional recovery, mental health, and quality of life of breast cancer survivors has become exceptionally important.

### 5.1 Antidepressant pharmacotherapy

Current clinical trials evaluating the efficacy of antidepressants for treating comorbid depressive disorders in breast cancer patients have yielded generally positive results. Agents such as amitriptyline, paroxetine, fluoxetine, mianserin, reboxetine, escitalopram, and bupropion have all demonstrated some therapeutic benefit. Among these, selective serotonin reuptake inhibitors (SSRIs) and serotonin-norepinephrine reuptake inhibitors (SNRIs) have become the mainstay pharmacological treatments for depression in breast cancer patients due to their established efficacy and favorable tolerability profile. Some SSRIs/SNRIs not only alleviate depressive symptoms but may also improve adverse effects associated with breast cancer treatment (85). Compared to traditional agents, while some newer antidepressants may not directly mitigate the side effects of breast cancer therapy, they exhibit unique advantages in managing specific symptoms. For instance, mirtazapine possesses antiemetic and gastroprokinetic effects, which can effectively improve cancer-related anorexia, thereby helping to maintain nutritional status and quality of life (86). Furthermore, sexual dysfunction is relatively common in breast cancer patients receiving endocrine therapy, and bupropion has shown potential for improving sexual function in this population, providing a basis for its individualized use in patients with comorbid mood issues (87).

Drug-drug interactions are a critical consideration. Tamoxifen is one of the most commonly used endocrine therapies for patients with hormone receptor (HR)-positive breast cancer. The cytochrome P450 2D6 (CYP2D6) enzyme is essential for converting tamoxifen, a prodrug, into its active metabolite, endoxifen. Any drug that inhibits CYP2D6 may reduce endoxifen plasma concentrations, potentially compromising tamoxifen’s efficacy in preventing recurrence. One study found that breast cancer patients using paroxetine or fluoxetine had approximately 50% lower endoxifen levels compared to normal patients (88). Another study involving 2,430 women receiving tamoxifen combined with a single SSRI found a significantly increased risk of breast cancer mortality associated with the concurrent use of paroxetine, whereas no such risk was found with other antidepressants (89). However, a large study of 16,887 breast cancer survivors after tamoxifen treatment found similar recurrence rates between antidepressant users and non-users, with no significant difference in recurrence rates among various antidepressants, including paroxetine and fluoxetine (90). Similarly, a large systematic review of 15 studies involving nearly 100,000 patients found that concomitant use of antidepressants (including paroxetine) and tamoxifen did not adversely affect clinical outcomes (91). Despite recent large-scale studies tending to be reassuring, based on pharmacological mechanisms and some clinical associations, current guidelines still tend to recommend cautious avoidance of potent CYP2D6 inhibitors—particularly paroxetine, fluoxetine, and bupropion—in patients taking tamoxifen. Such recommendations would provide clearer guidance for clinical decision-making.

Furthermore, researchers are developing novel antidepressants targeting systems such as glutamate/GABA, the HPA axis pathway, opioid receptors, brain-derived neurotrophic factor (BDNF), glial cell line-derived neurotrophic factor (GDNF), and histone modifications (92). These new-generation antidepressant drugs may provide additional options for managing the comorbidity of depression and breast cancer.

### 5.2 Psychosocial support therapies

Breast cancer patients commonly experience Fear of Cancer Recurrence or Progression (FCR) throughout their disease trajectory (93), a significant psychosocial factor that can induce or exacerbate depressive symptoms. The comprehensive application of established psychotherapeutic approaches—such as Cognitive Behavioral Therapy (CBT), supportive psychotherapy, Interpersonal Therapy (IPT), and psychodynamic therapy (PDT)—can not only help alleviate depressive symptoms in these patients but also enhance their ability to cope with the illness, improve quality of life, and increase treatment adherence.

A meta-analysis of 13 randomized controlled trials confirmed that CBT significantly reduces depression scores in post-operative breast cancer patients (94). The mechanism involves helping patients identify and restructure maladaptive cognitions related to disease recurrence and changes in body image, while also fostering adaptive behavioral patterns. Furthermore, supportive psychotherapy provides a safe space for emotional expression, helping to reduce feelings of isolation, whereas IPT, focusing on role transitions and improving communication patterns, offers unique value in maintaining social support. Another study reviewing psychological interventions for patients with metastatic breast cancer, which included 16 randomized controlled trials, found that psychotherapy significantly improved psychological distress, coping ability, and pain in these patients, though it did not show a clear benefit for survival (95). This suggests its effects may be more pronounced on quality of life rather than biological outcomes. Similarly, another meta-analysis of 27 randomized controlled trials (involving 7,742 participants; 3,880 in psychoeducation groups and 3,862 in control groups) found that, compared to the control group, psychoeducation had no significant effect on adherence to diagnostic procedures or medical treatment (*p* = 0.16) but did significantly reduce anxiety (*p* = 0.04) and improve quality of life (*p* < 0.01) (96). These findings suggest that the primary value of psychoeducation lies in its capacity to alleviate psychological distress and enhance coping strategies​ through mechanisms of emotional regulation and cognitive restructuring, thereby yielding positive effects on quality of life, rather than directly altering biomedical outcomes or treatment adherence.

**Figure 5:**
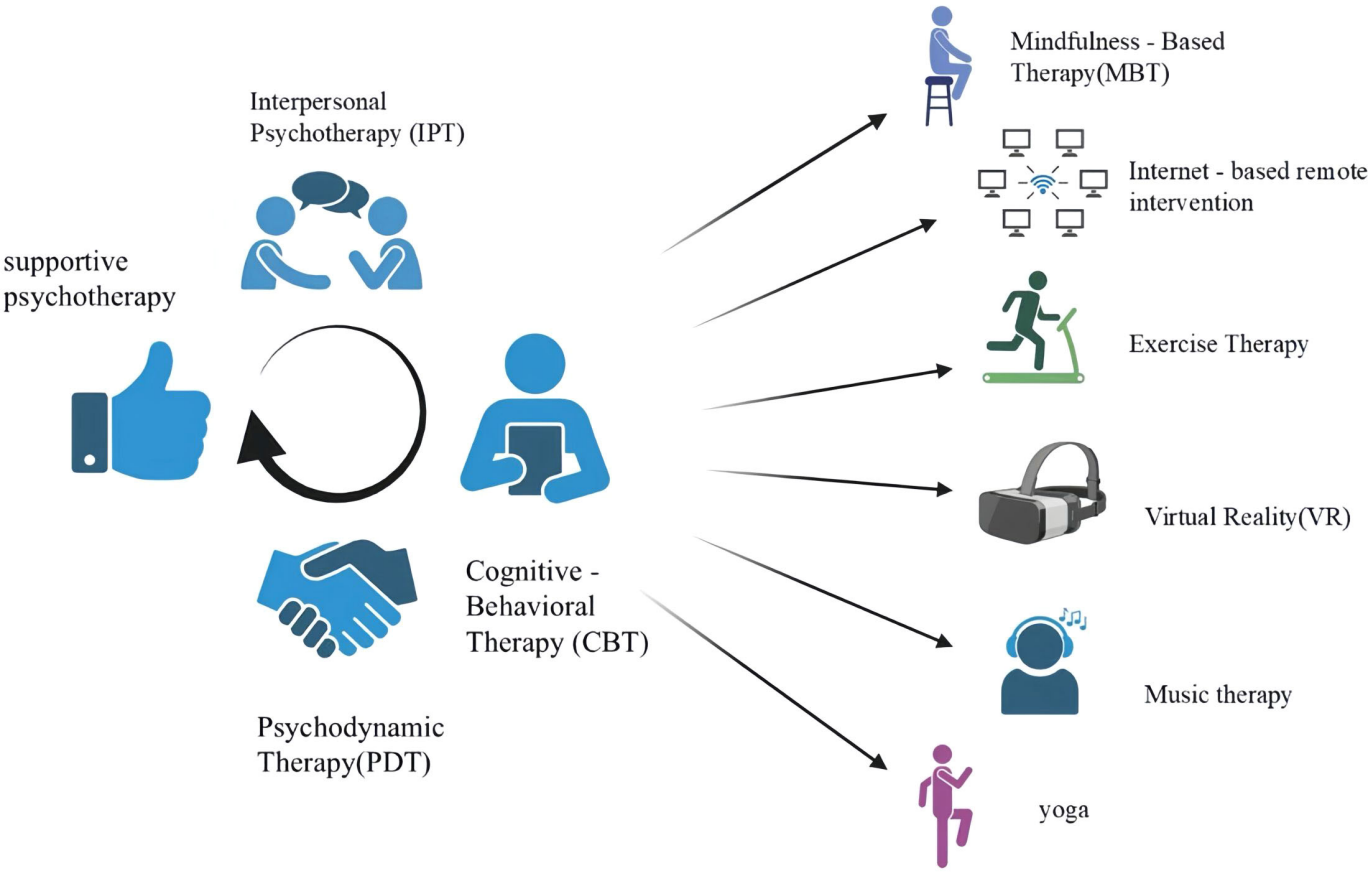
Application of multimodal and cross-domain psychological interventions in comorbid depression and breast cancer, integrating classic psychotherapies with mindfulness-based approaches, internet-based remote interventions, exercise, virtual reality, music therapy, and yoga.

Beyond traditional therapies, interventions integrating mind-body regulation techniques show broad application potential. One study investigating the impact of music therapy on anxiety, depressive symptoms, pain levels, and quality of life in breast cancer patients—pooling data from 10 studies involving 593 patients—showed that music intervention effectively alleviated anxiety symptoms (SMD: -2.12, 95% CI: -3.17 to -1.07), depressive symptoms (SMD: -0.77, 95% CI: -1.47 to -0.07), and pain levels (SMD: -3.47, 95% CI: -6.45 to -0.48), though the improvement in quality of life was not statistically significant (SMD: -0.07, 95% CI: -0.48 to 0.34) (97). Another study found that mindfulness yoga, as an adjunct to psychotherapy, effectively alleviated psychological stress caused by the disease and chemotherapy, reducing anxiety and depressive symptoms (98). For early-stage breast cancer patients undergoing adjuvant chemotherapy, mindfulness yoga intervention positively impacted anxiety and depression, enhancing psychological adjustment. Additionally, a study evaluating the efficacy of Virtual Reality (VR) for managing chemotherapy-related psychological distress in breast cancer patients, compared to music therapy, suggested that VR’s more immersive experience could fully engage patients in a virtual world, thereby providing more effective distraction and significantly reducing anxiety symptoms during chemotherapy (99). See **Figure 5**.

Psychological interventions have demonstrated efficacy not only in ameliorating emotional distress and improving quality of life but also in mitigating cancer-related fatigue and pain to a certain extent, all without the risk of drug-drug interactions. Consequently, integrating psychosocial support into routine oncology care pathways is an indispensable component for optimizing the long-term physical and psychological health outcomes of breast cancer survivors.

### 5.3 Other therapeutic approaches

According to Traditional Chinese Medicine (TCM) theory, the core pathogenesis of breast cancer-related depression (BCRD) is “Liver Qi Stagnation”. Treatment emphasizes soothing the liver to relieve stagnation and regulating Qi and blood. Recent research indicates that Chaihu Shugan San can simultaneously improve depression-like behaviors and inhibit tumor growth by modulating the “IL-17/NF-κB-microglial polarization” axis. The mechanisms involve repairing the blood-brain barrier, increasing hippocampal 5-HT and DA levels to approximately 90% of normal values, and reducing serum corticosterone and ACTH concentrations by about 40% (100).

Secondly, neuromodulation techniques achieve antidepressant effects by targeted modulation of brain network function. Physical therapies such as repetitive transcranial magnetic stimulation (rTMS) and transcranial direct current stimulation (tDCS) are gradually being applied to patients with comorbid breast cancer and depression. These techniques primarily work by modulating functional connectivity in the prefrontal-limbic circuit, thereby correcting HPA axis dysfunction and regulating the balance of the monoaminergic neurotransmitter system (101, 102).

Advances in nanotechnology offer novel strategies for precise targeted intervention in the comorbidity of depression and breast cancer. One study constructed ketamine-loaded lipid nanoparticles (LNP@Ket). By activating the nuclear factor erythroid 2-related factor 2 (Nrf2) pathway in astrocytes, these nanoparticles enhanced the activity of antioxidant enzymes such as heme oxygenase-1 (HO-1) and NAD(P)H quinone dehydrogenase 1 (NQO-1), and inhibited the release of pro-inflammatory factors like iNOS and TNF-α, thereby ameliorating anxiety and depressive behaviors in a mouse model of breast cancer. Single-cell transcriptome analysis confirmed that this system improves drug targeting to the central nervous system while reducing systemic side effects. This precise delivery strategy provides a direction for modifying traditional drugs (e.g., antidepressants) or natural active components (e.g., TCM extracts), potentially overcoming the blood-brain barrier and improving bioavailability (103).

**Figure 6:**
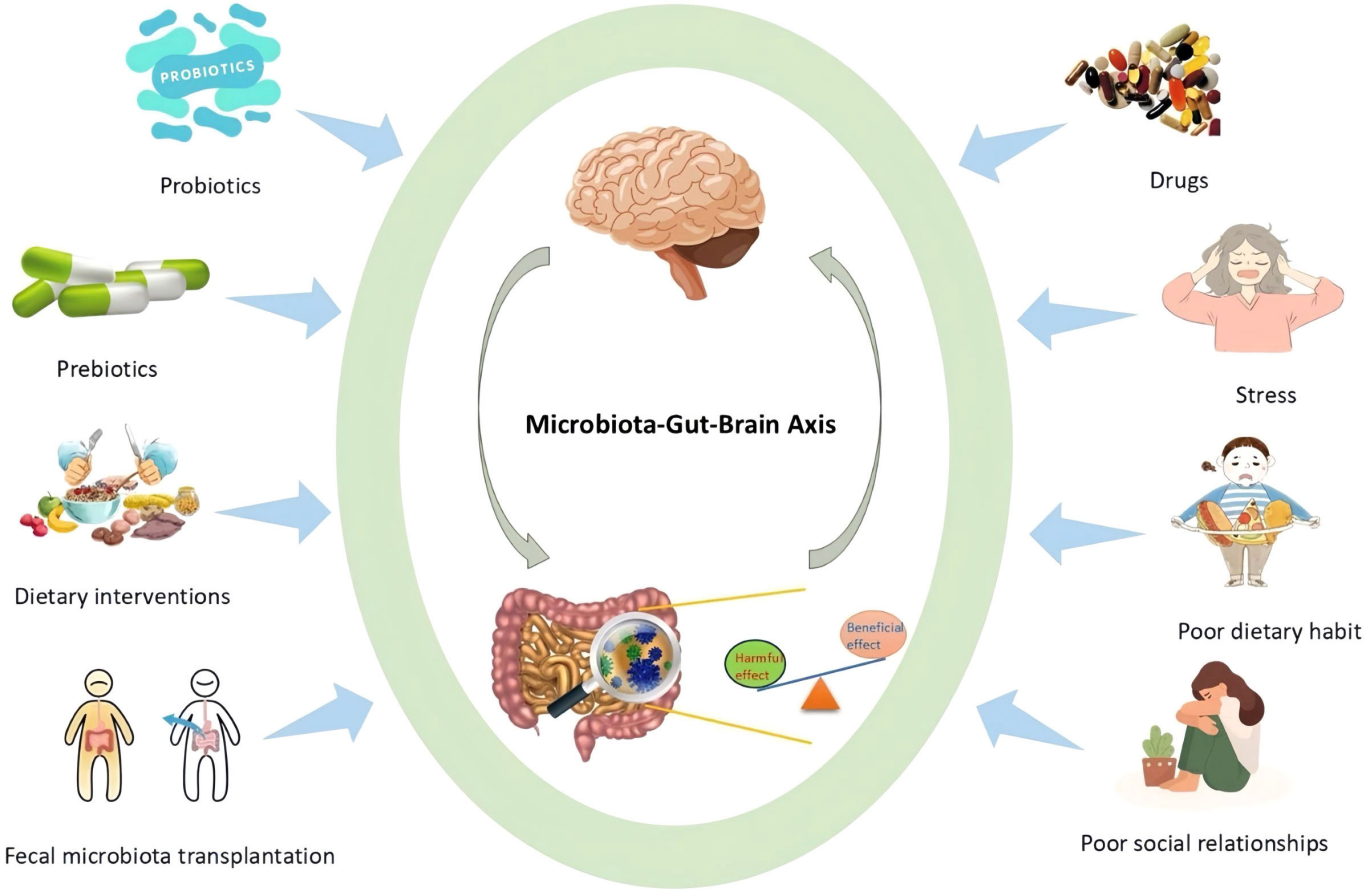
The relationship between gut microbiota dysbiosis and the central nervous system in patients with depression and the treatment methods.

The gut microbiota influences breast cancer progression and mood regulation directly or indirectly through metabolites, neurotransmitters, and immune-inflammatory pathways. Therefore, supplementing with specific prebiotics/probiotics can improve microbiota-gut-brain axis function. Strains such as Lactobacillus casei and BifidobacteriumBb-12 can balance tryptophan metabolism, increase serotonin synthesis, and mediate antidepressant effects via the vagus nerve (104). Research shows that fecal microbiota transplantation (FMT) successfully reversed microbiota dysbiosis and behavioral abnormalities in a mouse model of depression, a mechanism related to the restoration of the intestinal barrier and reduction of plasma LPS levels (105). Similarly, dietary patterns can modulate gut-brain axis signaling by altering microbiota structure and metabolites. Notably, a diet rich in dietary fiber promotes the fermentation by Bifidobacterium and Lactobacillusto produce short-chain fatty acids (SCFAs) like butyrate. Butyrate not only helps maintain intestinal barrier integrity but also inhibits the NF-κB pathway, reduces levels of pro-inflammatory cytokines such as IL-6 and TNF-α, and alleviates neuroinflammation (106). See **Figure 6**.

Emerging therapies—including Traditional Chinese Medicine approaches for soothing liver qi and relieving depression, neuromodulation techniques, nano-targeted drug delivery systems, and gut microbiota modulation—offer multidimensional and cross-disciplinary potential strategies for the comprehensive management of BCRD. A common feature of these interventions lies in their ability to regulate immune-inflammatory responses, neurotransmitter systems, and brain–body network functions, thereby achieving dual benefits for both emotional well-being and tumor control. However, current research efforts have been largely confined to preclinical studies or small-scale early-phase clinical trials, with a notable lack of high-quality clinical evidence derived from large-sample randomized controlled designs. Future investigations should prioritize multicenter, prospective clinical studies to validate the long-term efficacy, safety, and feasibility of these approaches, thereby facilitating their translation from bench to bedside.

## 6 Conclusions and perspectives

This review summarizes the epidemiological characteristics, complex bidirectional associations, and underlying multi-system interactive mechanisms of the comorbidity between depression and breast cancer. It encompasses various levels, from neuroendocrine dysregulation and immune suppression to neuroinflammation and gut-brain axis disruption. Depression promotes the initiation and progression of breast cancer primarily through the overactivation of the HPA axis, leading to persistently elevated glucocorticoid levels, which subsequently cause dysregulation of the sex hormone axis, prolactin imbalance, and insulin resistance. Concurrently, the depressive state inhibits the function of immune cells like T cells and NK cells, promotes the release of pro-inflammatory cytokines, and impairs immune surveillance, thereby creating a favorable environment for tumor growth. Regarding treatment, interdisciplinary integrated intervention strategies—including careful medication selection, diverse psychotherapies, and emerging approaches such as traditional Chinese medicine, neuromodulation techniques, and gut microbiota regulation—demonstrate greater potential than single-modality therapies. Overall, while current research on depression and breast cancer comorbidity has achieved remarkable advances, future endeavors still face multifaceted challenges and opportunities in deepening mechanistic insights and refining clinical management strategies.

The current understanding of the mechanisms underlying the depression-breast cancer comorbidity is largely based on macroscopic associations. Future research needs to leverage advanced technologies to delve into microscopic mechanisms and causal relationships. Multi-omics Integration and Precision Medicine:​ Single-cell transcriptomics, spatial proteomics, and metabolomic flux analysis can be employed to precisely characterize the dynamic alterations and interaction networks of distinct cell types—such as tumor-associated macrophages, T-cell subsets, and cancer-associated fibroblasts—within the breast cancer microenvironment under chronic stress conditions. Particular emphasis should be placed on elucidating how specific signaling pathways (e.g., glucocorticoid–GR signaling and the IDO1-kynurenine pathway) orchestrate gene expression and functional phenotypes in defined cellular contexts, thereby directly linking psychological stress to tumor biology. Focus on Epigenetic Regulation:​ Chronic stress-induced hormones and inflammatory cytokines can trigger alterations in genomic DNA methylation, histone modifications, and non-coding RNA expression. Future investigations should focus on key genes(such as BDNF,NEGR1,CYP7B1)—and their regulatory networks, to explore whether these epigenetic changes constitute a “molecular memory” linking early-life adversity, depression susceptibility, and breast cancer risk, thereby identifying novel, measurable, and controllable targets for intervention. Developing Multi-dimensional Biomarker Profiles:​ Integrating clinical information, molecular biomarkers, and neuroimaging data to construct predictive models using AI and machine learning. This aims to early identify high-risk patients and provide a basis for individualized treatment.

Long-term mental health and health equity for breast cancer survivors:​ with the improving survival rates of breast cancer, research should prioritize survivors’ long-term psychosocial adaptation, fear of cancer recurrence (FCR), cancer-related fatigue, cognitive decline, and social reintegration, while developing sustainable psychosocial support models. Concurrently, disparities in illness experiences across socioeconomic status, cultural backgrounds, and age groups should be addressed by exploring low-cost, highly accessible interventions based on mobile health (mHealth) platforms (e.g., smartphone-delivered self-help psychological training and community online support groups), thereby advancing global health equity.
